# Unveiling the Unpredictable in Parkinson’s Disease: Sensor-Based Monitoring of Dyskinesias and Freezing of Gait in Daily Life

**DOI:** 10.3390/bioengineering11050440

**Published:** 2024-04-29

**Authors:** Alessandro Zampogna, Luigi Borzì, Domiziana Rinaldi, Carlo Alberto Artusi, Gabriele Imbalzano, Martina Patera, Leonardo Lopiano, Francesco Pontieri, Gabriella Olmo, Antonio Suppa

**Affiliations:** 1 Department of Human Neurosciences, Sapienza University of Rome, 00185 Rome, Italy; alessandro.zampogna@uniroma1.it (A.Z.); martina.patera@uniroma1.it (M.P.); 2 IRCCS Neuromed Institute, 86077 Pozzilli, IS, Italy; 3 Data Analytics and Technologies for Health Lab (ANTHEA), Department of Control and Computer Engineering, Politecnico di Torino, 10129 Turin, Italy; luigi.borzi@polito.it (L.B.); gabriella.olmo@polito.it (G.O.); 4 Department of Neuroscience, Mental Health and Sense Organs (NESMOS), Sapienza University of Rome, 00189 Rome, Italy; domiziana.rinaldi@uniroma1.it (D.R.); francesco.pontieri@uniroma1.it (F.P.); 5 Department of Neuroscience “Rita Levi Montalcini”, University of Turin, 10126 Torino, Italy; caartusi@gmail.com (C.A.A.); gabri991@gmail.com (G.I.); leonardo.lopiano@unito.it (L.L.); 6 Neurology 2 Unit, A.O.U, Città della Salute e della Scienza di Torino, 10126 Torino, Italy

**Keywords:** Parkinson’s disease, dyskinesias, freezing of gait, wearable sensors, long-term monitoring

## Abstract

Background: Dyskinesias and freezing of gait are episodic disorders in Parkinson’s disease, characterized by a fluctuating and unpredictable nature. This cross-sectional study aims to objectively monitor Parkinsonian patients experiencing dyskinesias and/or freezing of gait during activities of daily living and assess possible changes in spatiotemporal gait parameters. Methods: Seventy-one patients with Parkinson’s disease (40 with dyskinesias and 33 with freezing of gait) were continuously monitored at home for a minimum of 5 days using a single wearable sensor. Dedicated machine-learning algorithms were used to categorize patients based on the occurrence of dyskinesias and freezing of gait. Additionally, specific spatiotemporal gait parameters were compared among patients with and without dyskinesias and/or freezing of gait. Results: The wearable sensor algorithms accurately classified patients with and without dyskinesias as well as those with and without freezing of gait based on the recorded dyskinesias and freezing of gait episodes. Standard spatiotemporal gait parameters did not differ significantly between patients with and without dyskinesias or freezing of gait. Both the time spent with dyskinesias and the number of freezing of gait episodes positively correlated with the disease severity and medication dosage. Conclusions: A single inertial wearable sensor shows promise in monitoring complex, episodic movement patterns, such as dyskinesias and freezing of gait, during daily activities. This approach may help implement targeted therapeutic and preventive strategies for Parkinson’s disease.

## 1. Introduction

Parkinson’s disease (PD) is a neurodegenerative disorder characterized by a wide range of motor and non-motor symptoms [1]. Among the most disabling manifestations of the disease are certain motor disorders that manifest abruptly and unpredictably, such as dyskinesias and freezing of gait (FOG). Dyskinesias are a phenomenon characterized by involuntary movements that can manifest as writhing, twisting, or jerking motions in any body segment, typically arising from the complex interplay between long-term dopaminergic therapy and disease progression [2]. Dyskinesias typically fluctuate throughout the day, sometimes in an unpredictable manner [2,3]. By causing sudden shifts in posture and weight distribution, they can lead to unexpected falls in any direction [3]. Concerning FOG, this entails sudden and transient episodes of immobility during walking, with a paroxysmal interruption of stride and/or marked reduction in forward foot progression [4,5]. It is heavily influenced by emotional, attentional, and environmental factors, so it often does not manifest under medical observation but predominantly occurs at home during daily activities [4].

The unpredictability and multifactorial nature of dyskinesias and FOG make it challenging to identify and manage these disorders solely through hospital clinical assessments and self-reports. Indeed, these phenomena may not occur during the limited observation period of the medical visit. Furthermore, patients and caregivers often fail to accurately recognize dyskinesias and FOG or may not be fully aware of them, thereby limiting the utility of medical history collection and the use of home-based diaries [6,7]. Accordingly, it would be advantageous to have a non-invasive technology capable of accurately identifying and quantifying these paroxysmal events. This approach would allow us to properly assess the occurrence and severity of dyskinesias and FOG and implement targeted interventions to reduce patients’ risk of falls and injuries.

The automatic recognition of complex motor patterns is now facilitated by innovative wearable technologies designed for the long-term and non-invasive monitoring of movements in young [8] and elderly healthy subjects [9], as well as patients with PD [10,11,12,13,14,15,16]. These technologies can incorporate advanced machine-learning algorithms to identify several motor disorders in PD, including postural instability [9,17,18], bradykinesia [19,20,21], dyskinesias, and FOG [22,23]. However, while high diagnostic performances have been demonstrated in laboratory settings under supervised conditions, only a narrow number of studies have assessed dyskinesias or FOG using wearable sensors in ecological, unsupervised environments [24,25,26,27,28,29]. Most authors used inertial sensors to monitor dyskinesias by simulating daily scenarios, which do not completely reflect ecological settings [25,28,30,31]. Indeed, in these studies, patients were asked to execute pre-defined motor tasks under medical observation during a relatively short monitoring period, spanning from 30 min to 4 h [25,28,30]. Accordingly, despite high diagnostic performances in identifying dyskinesias, these recordings failed to capture the typical day of Parkinsonian patients. Other authors monitored patients with PD for a prolonged time (i.e., 7 days) by using wrist- or waist-worn inertial sensors [32,33]. However, the adopted systems demonstrated a notably low sensitivity (i.e., 0.38) in detecting dyskinesias [32] as well as a weak agreement between sensor-based data and clinical scores [33], thus limiting their potential practical application. Concerning FOG, several authors performed long-term monitoring of this disorder through inertial sensors [27,34,35,36,37]. For instance, Denk et al. [34] investigated the accuracy of a smartphone and two inertial sensors in evaluating FOG compared to standardized clinical tools, demonstrating the high value of their combination. However, this study included a small group of subjects (i.e., 28 patients), and the average duration of monitoring was only 4 h per day [34]. A similar approach was also adopted by other authors with the primary aim of validating wearable sensors, providing limited practical clinical implications [35,36,37]. Overall, previous studies on dyskinesias and FOG in PD mostly relied on the use of multiple wearable sensors (potentially impractical for routine use), small patient samples, and short monitoring periods, ultimately failing to lead to valid solutions for clinical implementation [38]. Moreover, these studies did not compare spatiotemporal gait parameters in patients with and without dyskinesias and/or FOG in free-living situations nor did clarify whether patients with these paroxysmal disorders have prominent abnormalities in spatiotemporal gait parameters, as suggested by previous laboratory-based evaluations [39,40].

In this context, our main research question focused on the feasibility of unobtrusively identifying individuals with and without dyskinesias and FOG during daily activities, along with examining potential differences in spatiotemporal gait parameters among these patient subgroups in real-world settings. Accordingly, we continuously monitored a large cohort of patients with PD, with and without dyskinesias and/or FOG, by using a single wearable sensor for an extended period. We adopted a validated and approved class IIA medical device, incorporating ad hoc machine-learning algorithms for the recognition of dyskinesias and FOG [41]. We evaluated the psychometric properties of the wearable sensor. Furthermore, we examined spatiotemporal gait parameters in subgroups of patients, with and without dyskinesias and/or FOG, matching subjects according to demographic and clinical characteristics. Our first hypothesis was that continuous monitoring of patients with a single wearable sensor would demonstrate good diagnostic performance in recognizing individuals with and without dyskinesias and/or FOG. Furthermore, we also hypothesized that patients with and without dyskinesias or FOG would exhibit different spatiotemporal gait parameters when recorded during free-living, unsupervised situations. To this aim, unlike prior studies, the novelty of our measurements consists of the assessment of the diverse practical implications of sensors in the clinical management of dyskinesias and FOG in PD in addition to the evaluation of the performance of the wearable system. Lastly, by examining specific spatiotemporal gait parameters, we enhanced our study with pathophysiological insights that could increase our understanding of dyskinesias and FOG in PD.

## 2. Materials and Methods

This cross-sectional study followed the guidelines provided in the “Strengthening the Reporting of Observational Studies in Epidemiology” (STROBE) document, as detailed in Supplementary Materials S1.

### 2.1. Subjects

Patients with PD were longitudinally enrolled at the movement disorder outpatient clinics of the Sapienza University of Rome and the University of Turin (Italy) between January 2023 and February 2024. The inclusion criteria for the study encompassed diagnosis of idiopathic PD based on current consensus criteria [1]; Hoehn and Yahr (H&Y) between 1.5 and 4; PD duration of at least 3 years; and chronic therapy with L-Dopa ± other dopaminergic drugs. Exclusion criteria included diagnosis of possible or probable atypical parkinsonism; inability to walk independently; and comorbidities potentially affecting gait (e.g., neurological conditions other than PD, orthopedic and/or rheumatologic issues). All participants were regularly followed up by an expert in movement disorders and classified as patients with (PD-Dys) and without dyskinesias (PD-nDys) based on the use of the Unified Parkinson’s Disease Dyskinesia Rating Scale (UDysRS) (score ≥ 1 at item 1) and the direct observation of dyskinesias on at least one visit. Likely, the classification of participants as patients with (PD-FOG) and without FOG (PD-nFOG) was based on the clinical diagnosis of FOG through the direct observation of the disorder on at least one visit and using the FOG-Questionnaire. Before the long-term gait monitoring using wearable sensors at home, all patients underwent comprehensive clinical evaluations conducted by movement disorder experts in the outpatient clinic. These evaluations involved the administration of standardized scales, including the following: H&Y; Movement Disorders Society—Unified Parkinson’s Disease Rating Scale (MDS-UPDRS) parts III–IV; UDysRS parts III-IV; Wearing-OFF Questionnaire-19 (WOQ-19); FOG-Questionnaire; Montreal Cognitive Assessment (MoCA); Frontal Assessment Battery (FAB); Beck Depression Inventory (BDI); and Beck Anxiety Inventory (BAI). A follow-up clinical visit was conducted upon the return of the wearable sensor following the period of continuous monitoring at home. The clinical evaluations were conducted during the ON state of therapy (1 h after L-Dopa intake), reflecting prevalent clinical conditions at home during daily activities. The Levodopa equivalent daily doses (LEDDs) were calculated for each patient using standardized protocols [42]. No participants were receiving other neuropsychiatric medications possibly affecting gait during the study period.

All participants provided written informed consent, and the study protocol was approved by the Institutional Review Board of Sapienza University of Rome, Italy, in accordance with the principles outlined in the Declaration of Helsinki.

### 2.2. Study Protocol

**Figure 1:**
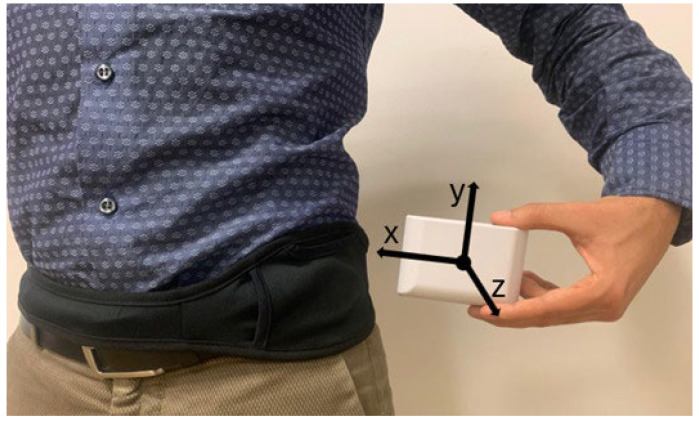
Wearable sensor (STAT-ON^TM^) position and axes orientation.

Each patient underwent long-term gait monitoring for a minimum of 5 days, at least 8 h per day, during daily life activities using a single wearable device (STAT-ON^TM^, Sense4Care, Barcelona, Spain) on the left side of the waist through an elastic belt (Figure 1). The device was positioned so that the x, y, and z axes of the embedded sensors represented the anterior, vertical (upward), and lateral (left) directions.

**Figure 2:**
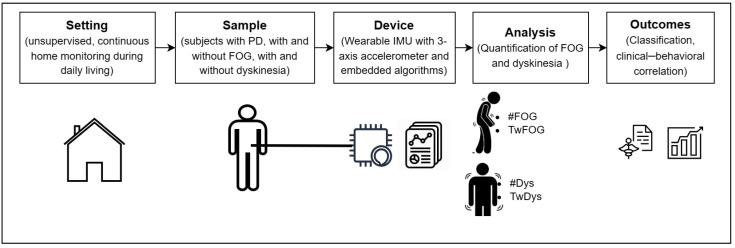
Experimental protocol of the study in patients affected by Parkinson’s disease (PD) with and without dyskinesias and/or freezing of gait (FOG). #FOG, number of FOG episodes; TwFOG, time spent with FOG; #Dys, number of dyskinesia periods; TwDys, time spent with dyskinesias.

Patients received thorough instructions on the proper use of the wearable sensor, including precise guidance on its placement, considering specific anatomical landmarks (e.g., left anterior–superior iliac spine). Patients were instructed to wear the sensor directly at home during waking hours and remove it during sleep or non-routine daily activities such as physical exercise, or extended travels by means of transportation. The wearable sensor was preconfigured by medical staff with the patient’s clinical data (i.e., H&Y, age, lower limb length measured from the left anterior–superior iliac spine to the ground). Therefore, patients were not required to perform any additional tasks except for wearing the sensor throughout the designated period, as the device would operate automatically for the entire battery life without the need for manual activation or deactivation. Figure 2 summarizes the experimental protocol, including the setting, study sample, adopted device, and resultant outcomes.

### 2.3. Wearable Sensor: Hardware

STAT-ON^TM^ is an inertial wearable medical device intended to continuously monitor motor manifestations in patients with PD during daily activities. The sensor measures 9 × 6.3 × 2.1 cm and weighs 86 g. Internally, the system is composed of two ultra-low triaxial nano-accelerometers, two microcontrollers, and a Bluetooth low-energy system, among other parts. The three-axis accelerometer has a full scale of ±6 g, a resolution of 12 bits, a sampling rate of 50 Hz, and a power consumption of 12 A. The device has a battery life of 7 days for continuous operation in normal conditions. STAT-ON^TM^ provides spatiotemporal gait parameters, such as step length, stride speed, and cadence, and can automatically detect motor patterns reflecting dyskinesias and FOG by using advanced machine-learning algorithms. Data are stored in an internal memory and can be downloaded by users (patients and clinicians) to any mobile phone that has the application provided by the manufacturer installed. The system has been certified as Medical Device Class IIa and has successfully passed the electromedical equipment tests, including those for home environment use. 

### 2.4. Wearable Sensor: Embedded Algorithms

The acceleration recordings are segmented into fixed-length timeframes of 3.2 s, overlapped by 50%. Gait detection is based on a support vector machine (SVM) classifier with a radial basis function. SVM represents a well-known and commonly used machine learning algorithm. It offers several advantages over other classic machine-learning algorithms, including memory-efficient implementation for on-device data processing [43], high generalization capability, and efficacy in handling non-linear decision boundaries [44]. For these reasons, SVM has been used in a large variety of applications, including activity [45], gait [46], and FOG recognition [47]. Two features are input to the SVM model, consisting of the energy contained within the frequency bands [0.1, 3] Hz and [0.1, 10] Hz [48]. If gait is detected, strides are identified by searching for the minima of the forward acceleration signal [49]. These latter represent the initial contact of the heel to the ground, also known as a “heel strike”. The two initial and two final strides are excluded from the analysis to increase consistency. From each stride detected, some gait-related parameters are extracted. Stride fluidity is computed as the energy content in the 0.1–10 Hz frequency band [48]. Step length is calculated based on the inverse pendulum model, which takes as input the leg length for correct estimation [50]. Cadence is computed as the ratio of the number of steps divided by the walking bout duration. This value is then multiplied by 60 to express the measure in steps/min. The 10 min dyskinesia output is positive when the energy content in the 1–4 Hz frequency band is above a certain threshold and that in the 0–20 Hz band is below a second threshold, for at least 6 sec. The second threshold is set to ensure that dyskinesias are searched when the patient is in a static position, thus reducing false positives due to voluntary movements [51]. The FOG detection algorithm is based on an SVM classifier with a radial basis function. The model is fed with specific temporal (e.g., increments, standard deviation, correlation among axes) and spectral (e.g., harmonic peaks, spectral skewness, and kurtosis) features extracted from the three-axis accelerometer signals [36]. The percentage of time spent with dyskinesia is computed every 10 min, while the number of FOG episodes and the time spent with FOG are calculated every minute, as previously reported [41].

### 2.5. Statistical Analysis

Descriptive statistics were used to describe the demographic and clinical characteristics of patients with PD by expressing continuous variables as median and interquartile range. The Mann–Whitney U-test was used to compare demographic and clinical features of subgroups of patients (i.e., PD-Dys vs. PD-nDys, PD-FOG vs. PD-nFOG), with a significance level of *p* < 0.05. Standardized effect sizes were computed by using Cohen’s d metric. Concerning the sample size, the significant methodological variability in existing literature made traditional power analysis challenging. To ensure robustness and applicability, we addressed this point by recruiting a larger cohort of patients compared to previous investigations [27,30,34,35]. Our sample size selection was thus based on participant enrollment from prior studies, supporting the robustness and reliability of our approach.

Data for each variable of interest were collected using wearable sensors. The primary outcome measures included the number of dyskinesia periods and the time spent experiencing dyskinesias, as well as the number of FOG episodes and the time spent with FOG. We selected these measures as study outcomes because they may reflect patients’ motor impairment extent and fall risk [52,53]. The cumulative distribution function (CDF) (which describes the probability of a variable having values less than or equal to x) was used to compare PD-Dys and PD-nDys as well as PD-FOG and PD-nFOG in terms of these primary outcomes. Then, to automatically and objectively identify subjects experiencing dyskinesias and/or FOG during activities of daily living, a binary classification task was set. For PD-Dys vs. PD-nDys classification, the input features comprised the number of dyskinesia periods, time spent with dyskinesias, and percent time spent with dyskinesias. For PD-FOG vs. PD-nFOG classification, the input features included the number of FOG episodes, time spent with FOG, and percent time spent with FOG. The latter measure was obtained by dividing the time spent with FOG by the total time walking. The input measures were normalized in the range 0–1 according to Equation (1), where *f*′ represents the normalized feature, *f* the original features, and *fmax* and *fmin* the maximum and minimum values, respectively:

(1) $$f’=\frac{f-fmin}{fmax+fmin}$$

Then, the performance of a logistic regression model in leave-one-subject-out validation was evaluated in terms of sensitivity, specificity, positive and negative predictive values (PPV and NPV, respectively), accuracy, and area under the curve (AUC).

A binary classification approach was also employed to differentiate patients experiencing both dyskinesias and FOG from those who were unaffected by each of the two disorders based on both dyskinesias and FOG measures.

The secondary outcome measures encompassed standard spatiotemporal gait parameters, including step length (m), stride speed (m/s), cadence (steps/min), and stride fluidity (m/s^2^). Given their substantial responsiveness to dopaminergic therapy, these spatiotemporal gait parameters adequately mirror the patient’s motor condition, potentially serving as a reliable surrogate for disease severity and stage [54,55]. Spatiotemporal gait parameters were calculated as average values within a 1 min timeframe, and their variance was also assessed. From the original recordings, zero-values and not available data were removed. In addition, parameters related to short walking periods (i.e., periods including less than 8 steps) were excluded from the analysis to reduce non-attentive or non-representative measures, reflecting potential confounders [27]. The Mann–Whitney U-test was used to compare spatiotemporal parameters between PD-Dys and PD-nDys as well as PD-FOG and PD-nFOG, with a significance level of *p* < 0.05. The effect size was calculated using Cohen’s d measure. To ensure comparability and minimize confounding factors, in addition to the implementation of rigorous data collection protocols and consistency in sensor placement, we conducted this analysis on a smaller number of patients from the entire enrolled cohort, allowing for the matching of subgroups based on age, disease duration, H&Y, MDS-UPDRS III, and MoCA. To this aim, an automated matching procedure was employed, which pairs each PD-Dys and PD-FOG with a control subject who is similar in terms of clinical and demographic characteristics, assigning equal weight to all measures. Accordingly, a stratified analytical approach was adopted to separately analyze subgroups of patients presenting dyskinesias or FOG.

Finally, Spearman’s correlation analysis was performed to assess possible clinical–behavioral associations between sensor-based measures and clinical scores. No missing data were encountered in the dataset. This was confirmed through a thorough review of the collected data before the analysis.

## 3. Results

Table 1 presents the demographic and clinical profiles of the entire PD patient cohort and subgroups categorized by the presence of dyskinesias and/or FOG, encompassing a total of 71 subjects. All enrolled patients meeting the study’s eligibility criteria completed the experimental procedures without any dropouts. Subjects were monitored for 6.7 ± 1.3 days (median: 6, IQR: 6–8 days). Following rigorous matching procedures based on demographic and clinical parameters, we identified a cohort of 48 individuals for comparing spatiotemporal gait parameters between PD-Dys and PD-nDys (consisting of 24 individuals each). Similarly, 48 subjects were selected to compare PD-FOG and PD-nFOG (also with 24 individuals in each). Table 2 summarizes the demographic and clinical characteristics of PD-Dys and PD-nDys, as well as PD-FOG and PD-nFOG, after matching procedures.

### 3.1. Dyskinesias

**Figure 3:**
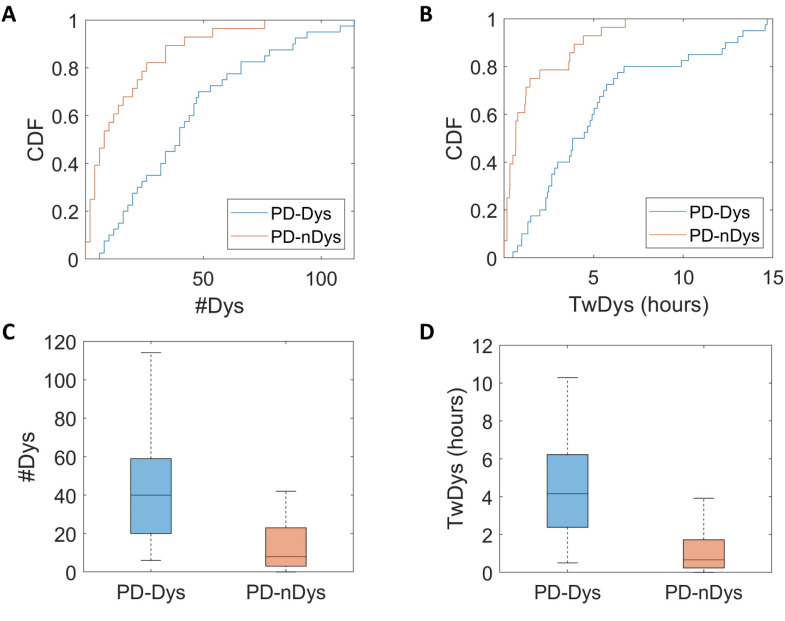
Cumulative distribution function (CDF) plots (**A**,**B**) and box plots (**C**,**D**) show a greater number of dyskinesia periods (#Dys) and time spent with dyskinesias (TwDys) in dyskinetic (PD-Dys) compared to non-dyskinetic Parkinsonian patients (PD-nDys).

The cumulative distribution function (CDF) showed that 57% of PD-nDys had zero detected dyskinesias, 82% had one period at most, and all of them had less than eight periods. Among PD-Dys, 10% of subjects had zero detected dyskinesia, 20% had one episode at most, and 70% of them had less than eight periods (Figure 3A,B). Both the number of dyskinesia periods (U = 192, *p* < 0.001, d = 1) and time spent with dyskinesias (U = 164, *p* < 0.001, d = 1) were statistically different between PD-Dys and PD-nDys (Figure 3C,D).

Regarding the classification of subgroups based on the time spent with dyskinesias, PD-Dys could be distinguished from PD-nDys with sensitivity 0.81, specificity 0.80, PPV 0.87, NPV 0.71, accuracy 0.81, and AUC 0.86.

When comparing step length, stride speed, stride fluidity, and cadence between PD-Dys and PD-nDys, the Mann–Whitney U-test did not show any significant differences (all *p* > 0.05 and d < 0.4) (Table 3).

Finally, the Spearman’s correlation test demonstrated that the time spent with dyskinesias significantly correlated with the MDS-UPDRS IV (r = 0.41, *p* < 0.001), UDysRS III (r = 0.52, *p* < 0.001) and IV (r = 0.52, *p* < 0.001), as well as LEDDs (r = 0.43, *p* < 0.001).

### 3.2. Freezing of Gait

**Figure 4:**
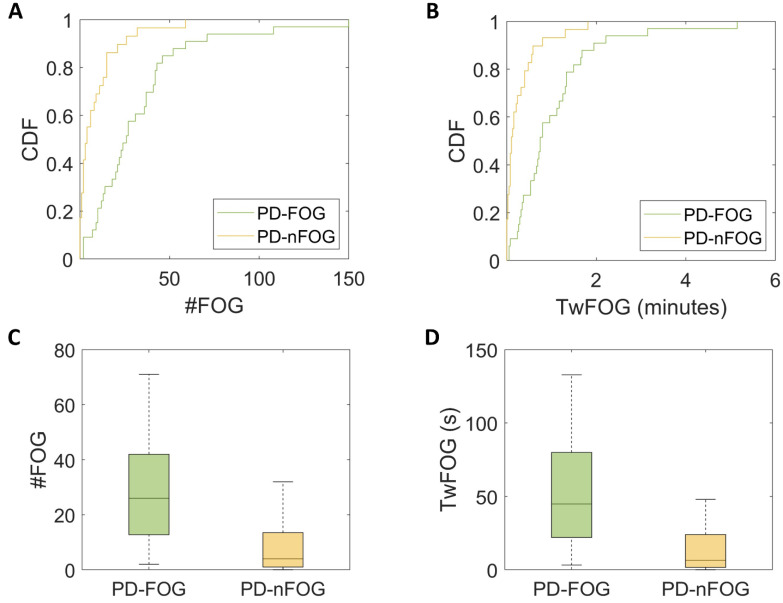
Cumulative distribution function (CDF) plots (**A**,**B**) and box plots (**C**,**D**) show a greater number of freezing of gait (FOG) episodes (#FOG) and time spent with FOG (TwFOG) in Parkinsonian patients with FOG (PD-FOG) and compared to those without FOG (PD-nFOG).

As shown by the CDF (Figure 4A,B), 59% of PD-nFOG had zero episodes detected, 83% had one episode at most, and all of them had less than seven episodes. By contrast, 12% of PD-FOG had zero episodes detected, 27% had one episode at most, and 51% of them had more than seven episodes. Both the number of FOG episodes (U = 127, *p* < 0.001, d = 0.9) and the time spent with FOG (U = 124, *p* < 0.001, d = 0.9) were statistically different between PD-FOG and PD-nFOG (Figure 4C,D). These findings did not change when normalizing the derived measures by the total time spent walking.

When classifying subgroups based on the number of FOG episodes and time spent with FOG, PD-FOG could be distinguished from PD-nFOG with the following performances: sensitivity 0.82, specificity 0.79, PPV 0.82, NPV 0.79, accuracy 0.81, and AUC 0.83.

Concerning spatiotemporal parameters of gait, no statistically significant differences in step length, stride speed, stride fluidity, and cadence, as well as the variance of these parameters, emerged between PD-FOG and PD-nFOG (all *p* > 0.05) (Table 3).

Lastly, Spearman’s correlation analysis showed that the number of FOG episodes was positively associated with scores of MDS-UPDRS IV (r = 0.54, *p* < 0.001), UDysRS III (r = 0.51, *p* < 0.001) and IV (r = 0.54, *p* < 0.001), WOQ19 (r = 0.48, *p* < 0.001), and LEDDs (r = 0.41, *p* = 0.001). In PD-FOG, neither the number of episodes (*p* = 0.29) nor the total duration of FOG (*p* = 0.21) showed a significant correlation with the FOG-Q. No significant correlations were found between clinical scores and the time spent with FOG.

### 3.3. Dyskinesias plus Freezing of Gait

When distinguishing patients concurrently experiencing dyskinesia and FOG (*n* = 30) from those unaffected by both these conditions (*n* = 28), the classification reached the following metrics: sensitivity of 0.83, specificity of 0.76, PPV of 0.83, NPV of 0.76, accuracy of 0.80, and an AUC of 0.92. Examining the individual contributions of features to the overall performance, the time spent with FOG presented the most robust outcome (AUC 0.87), followed by the number of FOG episodes (AUC 0.87) and the time spent with dyskinesias (AUC 0.83).

## 4. Discussion

In this study, we have continuously monitored a large cohort of patients with PD in ecologically valid environments, by using a validated and approved wearable sensor for an extended period. We have confirmed satisfactory psychometric properties of the wearable sensor in recognizing dyskinesias and FOG in patients with PD through dedicated machine-learning algorithms. Furthermore, we have provided novel findings regarding spatiotemporal gait parameters obtained during everyday activities in PD-Dys and PD-FOG.

By carefully adopting stringent inclusion criteria and methodological precautions, we have prevented several confounding factors possibly leading to misinterpretation. More in detail, we only included patients with idiopathic PD and we confirmed the presence of dyskinesias and FOG through direct observation of the disorder on at least one occasion. Moreover, to compare spatiotemporal gait parameters, we meticulously matched PD-Dys and PD-nDys, as well as PD-FOG and PD-nFOG, to avoid confounding arising from clinical variables influencing gait. Lastly, clear and precise instructions were provided for sensor placement to mitigate potential patient errors when using the device.

### 4.1. Dyskinesias

In line with clinical evidence indicating that dyskinesias are a motor complication of advanced disease [3], PD-Dys presented longer PD duration and greater severity of motor symptoms than PD-nDys. Advanced disease may pose a potential confounding factor in distinguishing dyskinesias from intentional gestures of daily life [56]. This task may be even more challenging when considering the broad spectrum of activities and their variability in unsupervised real-life conditions, as proposed by our study. Indeed, as the disease progresses, patients face increasing challenges in motor control, leading to voluntary movements that may deviate significantly from physiological motor patterns [57]. However, specific motor features, such as lower frequency components, irregular amplitude signals, and diminished coordination, may play a crucial role in differentiating dyskinesias from voluntary movements through kinematic analysis [56,58,59,60]. Despite the challenges associated with advanced disease, in our study, the wearable system has demonstrated moderate accuracy in distinguishing PD-Dys from PD-nDys, thus confirming our research hypothesis. The diagnostic performances of the wearable sensor were only slightly inferior to those previously reported in patients recorded under supervised conditions [28,61]. Moreover, in line with a previous study [30], clinical–behavioral correlations supported the robustness of sensor-based measures and their close association with oral dopaminergic therapy. The objective identification of dyskinesias in PD offers potential clinical advantages, given that this condition often requires a transition to infusion-based treatments (e.g., continuous intestinal infusion of Levodopa/Carbidopa gel or subcutaneous apomorphine) and/or surgical interventions (e.g., deep brain stimulation of the subthalamic nucleus or globus pallidus pars interna) [62]. Indeed, quantifying dyskinesias in daily life through the use of a single wearable sensor may facilitate the identification of patients who could benefit from invasive treatments and help manage therapeutic adjustments in the postoperative period [63].

When considering dyskinesias, the body position of the wearable sensor holds a certain importance [64,65]. Indeed, a sensor placed at the waist can provide information regarding trunk and lower limb dyskinesias but may not identify possible dyskinesias occurring in the upper limbs. Supporting this observation, previous authors demonstrated that the concordance between waist-worn sensor measurements and clinical assessments of dyskinesias notably increases when concentrating specifically on the trunk and lower limbs, as opposed to evaluating all body regions. [30]. Nonetheless, it should be considered that trunk and lower limb dyskinesias are likely of greater clinical interest as they have a more significant impact on static and dynamic postural control and, consequently, on the likelihood of falls [66]. Furthermore, given that the trunk is commonly affected in “peak-dose dyskinesias” (occurring when Levodopa is at its highest concentration in the body), while the lower limbs are implicated in “biphasic dyskinesias” (occurring during both the ascending and descending phases of medication effectiveness) [67], sensor-based monitoring could offer relevant insights on dyskinesias timing to optimize pharmacological treatment.

A further comment concerns the assessment of spatiotemporal gait parameters in PD-Dys. As dyskinesias disrupt the ability to walk smoothly and cohesively [68], measures such as step length and walking speed would be expected to be more severely impaired in PD-Dys than in PD-nDys [69]. In disagreement with our research hypothesis, we found comparable spatiotemporal gait parameters in clinically matched PD-Dys and PD-nDys, possibly underestimating the impact of dyskinesias on gait in PD. Several interpretations could be proposed on this issue: First, it is possible that the severity of patients’ dyskinesias was insufficient to significantly impair gait during the sensor-based recordings; alternatively, patients might have refrained from walking during periods of severe dyskinesias to minimize their risk of falls, thereby concealing the effects of involuntary movements on gait; lastly, it cannot be excluded that the sensitivity of a single wearable sensor may be inadequate to detect subtle changes in spatiotemporal gait parameters in real-world settings, where various environmental factors come into play [70]. Given the lack of prior studies comparing spatiotemporal gait parameters between PD-Dys and PD-nDys in both laboratory and home environments, we believe it is advisable to interpret this finding cautiously.

### 4.2. Freezing of Gait

Our PD-FOG cohort presented more severe disease characteristics compared to PD-nFOG, including longer disease duration and higher motor and non-motor impairment. This aligns with previous studies indicating that FOG is associated with advanced stages of PD, marked by more severe motor dysfunction, emotional impairment, and cognitive decline [71]. These findings emphasize the value of wearable sensors for objectively monitoring motor disorders in PD-FOG, as these patients may face greater challenges in accurately and reliably reporting their health status due to recall bias or inaccurate self-assessments [6,72]. Additionally, the greater clinical impairment in PD-FOG further underscores the need to minimize the number of devices used to enhance usability and reduce obtrusiveness [73]. Indeed, the reduced accuracy of single-sensor measurements compared to multiple devices is offset by a reduced wearability burden and increased long-term patient compliance [73]. In this context, placing the device on the waist or lower back may offer the right balance between low obtrusiveness and accurate recognition of FOG, as demonstrated by our findings and previous authors [73,74]. Accordingly, our methodological approach utilizing a single wearable sensor may offer greater convenience for implementation in daily clinical practice compared to previous studies that employed two or more devices for detecting FOG [27,34].

By stratifying patients based on sensor-based measures, in agreement with our research hypothesis, the number of FOG episodes and time spent with FOG allowed us to effectively differentiate PD-FOG from PD-nFOG with satisfactory diagnostic performances, both in terms of sensitivity and specificity. When looking at the number of FOG episodes, a lower frequency was observed in our study compared to similar research [27,34], potentially due to either a milder severity of the disorder in our patients or a reduced rate of false positives generated by our device. Also, the classification performance is notably lower compared to that observed in controlled laboratory environments, where the accuracy levels in FOG recognition are excellent [75,76]. However, it is essential to consider that, while in a laboratory setting patients are recorded under controlled conditions, long-term monitoring during routine daily activities introduces a high number of interfering factors that can impact patients’ gait, such as floor variations, environmental distractions, and variable task complexity [70]. Indeed, the performance of the adopted wearable system is fully comparable with that from previous at-home recordings of FOG in PD [36,77,78].

When comparing step length, stride speed, cadence, and stride fluidity, as well as their variance between PD-FOG and PD-nFOG, we did not find any significant differences, in contrast with what we initially hypothesized. This result apparently disagrees with some previous studies showing greater continuous gait abnormalities, such as shorter stride length and increased step-to-step variability, in PD-FOG than in PD-nFOG [39,40,75]. Moreover, our observations would also disagree with the pathophysiological hypothesis that suggests FOG in PD arises from a progressive deterioration of spatiotemporal gait parameters until reaching a threshold of impairment triggering the paroxysmal interruption of step (i.e., sequence effect) [79,80]. However, it should be considered that we monitored patients in real-life and unsupervised conditions, whereas studies reporting different gait parameters between PD-FOG and PD-nFOG were conducted in controlled laboratory environments [39,40]. It has been previously demonstrated that the assessment of mobility under supervised versus unsupervised conditions can yield markedly different results [81,82]. It is plausible that, in addition to environmental interfering elements, various attentional and emotional states significantly impact gait performances in free-living situations [83]. Therefore, it could be challenging to accurately measure subtle changes in spatiotemporal gait parameters in ecological settings. Indeed, consistent with our findings, other authors similarly found no differences in other spatiotemporal gait parameters between PD-FOG and PD-nFOG in home settings [27]. Overall, while the sensor accuracy in detecting subtle changes in spatiotemporal gait parameters may appear limited in PD, the system appears effective in identifying complex motor patterns like FOG. Our clinical–behavioral correlations further support this observation, also in line with previous findings [27,34]. Accordingly, future studies will need to explore alternative approaches for characterizing the gait signal in PD, recognizing that conventional methods for gait detection and stride segmentation may not be as efficacious to measure standard spatiotemporal gait parameters in unsupervised environments as they are in controlled laboratory settings.

While our study offers new valuable insights, it is important to also acknowledge some limitations. First, the absence of a gold standard tool for at-home use makes it challenging to directly assess the sensor performance in detecting dyskinesias and FOG in PD. This limitation is compounded by the inherently complex nature of these phenomena, making it difficult to establish a definitive benchmark. Moreover, the presence of the wearable sensor may have introduced a “Hawthorne effect”, potentially altering patients’ behaviors and thus affecting the ecological validity of the recorded data. These limitations underscore the need for further research to validate our findings and address these methodological challenges. Nonetheless, the study offers robust external validity and generalizability of the results. Indeed, strict inclusion criteria and careful research methodology were implemented to maximize the representativeness of participants compared to the reference population. Moreover, the highly heterogeneous and non-standardized home environment where patients were monitored suggests the potential adaptability of the methodological approach to various settings.

## 5. Conclusions

This study has demonstrated the potential to accurately recognize patients with PD experiencing dyskinesias and/or FOG in real-life settings, using a single unobtrusive wearable sensor embedding dedicated machine-learning algorithms. While highly sensitive in identifying complex motor patterns, this approach did not allow for the detection of possible changes in spatiotemporal gait parameters among patients with and without dyskinesias or FOG. Wearable sensors have undergone extensive laboratory testing, but their utilization in domestic environments remains limited, emphasizing the necessity for more real-life experiences to facilitate the transition of these tools from experimental to clinical settings. Overall, our findings offer the opportunity to improve the clinical care of patients with PD in advanced disease stages by accurately identifying individuals with motor complications and optimizing treatment strategies. Additionally, it opens up the prospect of implementing telemedicine procedures in patients affected by movement disorders.

## Figures and Tables

**Table 1:** Demographic and clinical features of patients with Parkinson’s disease.

	PD-ALL	PD-Dys	PD-nDys	PD-Dys vs. PD-nDys	PD-FOG	PD-nFOG	PD-FOG vs. PD-nFOG
Sex	56 M 15 F	27 M 13 F	27 M 1 F	U = 398.0 *p* = 0.002	27 M 6 F	23 M 6 F	U = 466.5 *p* = 0.406
Age (years)	69 (62.5–76)	67.5 (61–74)	73 (66–77.5)	U = 407.5 *p* = 0.029	67 (61.5–74)	71 (63–76)	U = 378.0 *p* = 0.079
Disease duration (years)	8.5 (5–12)	10.5 (7–13)	5.5 (4–8)	U = 240.5 *p* < 0.001	10 (7–13.2)	7 (4–10)	U = 225.5 *p* < 0.001
Hoehn and Yahr	2 (2–3)	2 (2–2.5)	2 (1.8–2)	U = 411.5 *p* = 0.015	2 (2–2.5)	2 (2--2)	U = 362.5 *p* = 0.023
MDS-UPDRS III ON	20.5 (16–30)	20.5 (15.5–29.5)	20.5 (17–31)	U = 505.5 *p* = 0.427	22.5 (15.5–31)	19.5 (15–25.5)	U = 369.0 *p* = 0.122
MDS-UPDRS IV	5 (1–9)	7.5 (5–12)	0.5 (0–3)	U = 148.5 *p* < 0.001	9 (5–12)	1 (0–5.2)	U = 121.5 *p* < 0.001
Unified dyskinesia rating scale-III	1 (0–5)	4 (2–9)	/	/	4 (1–9.2)	0 (0–1.5)	U = 168.0 *p* < 0.001
Unified dyskinesia rating scale-IV	1 (0–4)	3 (2–6)	/	/	3 (1–6)	0 (0–0.2)	U = 161.5 *p* < 0.001
Wearing-off questionnaire-19	4 (1–6.8)	5 (2.2–8)	0.5 (0–4.5)	U = 262.0 *p* < 0.001	6 (3.8–8.2)	1.5 (0–4.5)	U = 179.5 *p* < 0.001
Montreal cognitive assessment	25 (23–27)	25 (23–27)	25 (23–27)	U = 513.5 *p* = 0.468	25 (22.8–26)	25.5 (23–27)	U = 386.5 *p* = 0.137
Frontal assessment battery	15 (12–17)	15.5 (12–17)	15 (14–16)	U = 510.5 *p* = 0.354	14 (12–17)	16 (14–17)	U = 369.0 *p* = 0.088
Beck depression inventory	6.5 (4–10.5)	6 (4–10)	7 (4–11)	U = 414.0 *p* = 0.340	7 (4–10.2)	7 (4–12)	U = 368.5 *p* = 0.446
Beck anxiety inventory	8 (2–10.5)	9 (2.8–29)	7 (2–8)	U = 88.0 *p* = 0.140	16.5 (8–34)	7.5 (2.5–9.5)	U = 42.5 *p* = 0.065
Parkinson’s disease questionnaire-39	24.5 (15.5–38)	27 (17–38.8)	20 (13–37.2)	U = 365.5 *p* = 0.142	30.5 (23–40)	19.5 (13.5–37.5)	U = 258.5 *p* = 0.039
Freezing of gait questionnaire	4 (0–11)	9 (4–13)	/	U = 158.0 *p* < 0.001	10 (6.8–14)	/	/
Levodopa equivalent daily doses (mg)	850 (600–1220)	1125 (805–1350)	602 (500–752)	U = 251.5 *p* < 0.001	1170 (910–1587)	700 (503–865)	U = 195.5 *p* < 0.001

F, females; M, males; MDS-UPDRS, Movement Disorder Society—Unified Parkinson’s Disease Rating Scale; ON, under dopaminergic therapy; PD-ALL, entire cohort of patients with Parkinson’s disease; PD-Dys, patients with dyskinesia; PD-nDys, patients without dyskinesia; PD-FOG, patients with freezing of gait; PD-nFOG, patients without freezing of gait.

**Table 2:** Demographic and clinical features of matched patients with Parkinson’s disease.

	PD-Dys	PD-nDys	PD-Dys vs. PD-nDys	PD-FOG	PD-nFOG	PD-FOG vs. PD-nFOG
Sex	16 M 8 F	23 M 1 F	U = 204 *p* = 0.005	20 M 4 F	18 M 6 F	U = 264 *p* = 0.245
Age (years)	68 (64–74)	73 (66–77)	U = 220 *p* = 0.083	64 (56.5–70)	71 (62–75)	U = 202.5 *p* = 0.051
Disease duration (years)	8.5 (6–11)	6 (4–9)	U = 189 *p* = 0.053	9.5 (6–11)	7 (5–10)	U = 220 *p* = 0.081
Hoehn and Yahr	2 (2–2)	2 (2–2)	U = 250 *p* = 0.167	2 (2–2)	2 (2–2)	U = 255 *p* = 0.199
MDS-UPDRS III ON	19.5 (12.5–23.5)	20.5 (17–32)	U = 219 *p* = 0.166	19 (12.2–30.5)	19 (14.5–23.2)	U = 251 *p* = 0.387
MDS-UPDRS IV	6 (5–12.5)	0.5 (0–3)	U = 91 *p* < 0.001	10 (6–12.5)	2 (0–6)	U = 80 *p* < 0.001
Unified dyskinesia rating scale-III	3 (1–9)	/	/	3 (1–9)	0 (0–3)	U = 130 *p* < 0.001
Unified dyskinesia rating scale-IV	2 (1.5–6)	/	/	2.5 (1–5.5)	0 (0–1.5)	U = 118 *p* < 0.001
Wearing-off questionnaire-19	4 (2–5)	0.5 (0–4.5)	U = 150 *p* = 0.003	6 (4–9)	3 (0–5)	U = 107 *p* < 0.001
Montreal cognitive assessment	25 (22.5–26)	25 (23–27)	U = 237 *p* = 0.279	25 (21.5–26.5)	26 (24.2–27.8)	U = 207 *p* = 0.071
Frontal assessment battery	15 (12–17)	15 (14–16.8)	U = 256 *p* = 0.341	14 (12–17)	16 (14.2–17)	U = 194 *p* = 0.054
Beck depression inventory	6.5 (4–10.5)	7.5 (4–12)	U = 196 *p* = 0.281	8 (5.5–10.5)	8 (4–13.2)	U = 200 *p* = 0.397
Beck anxiety inventory	10 (8–29)	7.5 (3–9)	U = 38 *p* = 0.071	29 (16–34.5)	8 (4.2–10.8)	U = 13 *p* = 0.020
Parkinson’s disease questionnaire-39	22 (13.8–38.2)	21 (15.5–37.2)	U = 218 *p* = 0.485	33 (22.2–40.5)	20.5 (16–39)	U = 165 *p* = 0.123
Freezing of gait questionnaire	6 (3–11)	/	U = 82 *p* < 0.001	10 (6.5–13)	/	/
Levodopa equivalent daily doses (mg)	1050 (785–1240)	612 (500–752)	U = 138 *p* = 0.002	1125 (866–1607)	700 (55–955)	U = 142 *p* = 0.002

F, females; M, males; MDS-UPDRS, Movement Disorder Society—Unified Parkinson’s Disease Rating Scale; ON, under dopaminergic therapy; PD-ALL, entire cohort of patients with Parkinson’s disease; PD-Dys, patients with dyskinesia; PD-nDys, patients without dyskinesia; PD-FOG, patients with freezing of gait; PD-nFOG, patients without freezing of gait.

**Table 3:** Spatiotemporal gait parameters in Parkinsonian patients with (PD-FOG) and without FOG (PD-nFOG) as well as with (PD-Dys) and without dyskinesia (PD-nDys).

	PD-Dys	PD-nDys	PD-Dys vs. PD-nDys	PD-FOG	PD-nFOG	PD-FOG vs. PD-nFOG
Step length (m)	0.8 (0.7–0.8)	0.8 (0.7–0.8)	U = 274, *p* = 0.39 d = 0.10	0.8 (0.7–0.9)	0.8 (0.7–0.9)	U = 287, *p* = 0.496 d = 0.04
Stride speed (m/s)	0.5 (0.5–0.5)	0.5 (0.5–0.5)	U = 281, *p* = 0.447 d = 0.1	0.5 (0.5–0.6)	0.5 (0.5–0.5)	U = 257, *p* = 0.265 d = 0.02
Stride fluidity	8.1 (7–9.1)	7.3 (6.9–8.2)	U = 217, *p* = 0.073 d = 0.4	8 (7.3–9.3)	7.8 (7–8.5)	U = 261, *p* = 0.292 d = 0.2
Cadence	38.9 (37.9–40)	39.1 (37.6–40)	U = 271, *p* = 0.367 d = 0.05	38.9 (37.7–40.5)	39.1 (37.6–40)	U = 285, *p* = 0.479 d = 0
Std step length (m)	0.2 (0.2–0.2)	0.2 (0.2–0.2)	U = 234, *p* = 0.140 d = 0.4	0.2 (0.2–0.2)	0.2 (0.2–0.2)	U = 246, *p* = 0.196 d = 0.4
Std stride speed (m/s)	0.1 (0.1–0.1)	0.1 (0.1–0.1)	U = 284, *p* = 0.471 d = 0.1	0.1 (0.1–0.1)	0.1 (0.1–0.1)	U = 275, *p* = 0.398 d = 0.2
Std stride fluidity	1.5 (1.2–2)	1.4 (1.1–1.8)	U = 242, *p* = 0.174 d = 0.3	1.5 (1.3–2)	1.7 (1.3–1.9)	U = 272, *p* = 0.375 d = 0.1
Std cadence	5.3 (4.6–5.6)	5.4 (4.8–5.8)	U = 256, *p* = 0.258 d = 0.2	5.5 (4.8–6)	5.4 (4.9–5.7)	U = 249, *p* = 0.214 d = 0.3

PD-Dys, patients with dyskinesia; PD-nDys, patients without dyskinesia; PD-FOG, patients with freezing of gait; PD-nFOG, patients without freezing of gait; Std, standard deviation.

## Data Availability

The dataset used in this study can be obtained from the corresponding author upon reasonable request. Access to sensitive data will be restricted to preserve privacy.

## Supplementary Materials

The following supporting information can be downloaded at: https://www.mdpi.com/article/10.3390/bioengineering11050440/s1, Supplementary Material S1: STROBE checklist.
